# Work among the people of the Okapa area from 1996 to the present

**DOI:** 10.1098/rstb.2008.4035

**Published:** 2008-11-27

**Authors:** Jerome T. Whitfield

**Affiliations:** MRC Prion Unit and Department of Neurodegenerative Disease, UCL Institute of Neurology, National Hospital for Neurology and NeurosurgeryQueen Square, London WC1N 3BG, UK; Centre for International Health, ABCRCShenton Park Campus, Curtin University, GPO Box U1987, Perth, WA 6845, Australia; Papua New Guinea Institute of Medical ResearchPO Box 60, Goroka, EHP 441, Papua New Guinea

I have conducted fieldwork on kuru, with colleagues from the MRC Prion Unit and the Papua New Guinea Institute of Medical Research (PNGIMR), since 1996. During this time, the fieldwork has been dependent on the support of the PNGIMR, and I thank the staff and the past and present Directors, Michael Alpers, John Reeder and Peter Siba, for their support. Michael's vast knowledge and experience of Papua New Guinea has been invaluable and inspirational. I also thank my Papua New Guinean friends whose counsel has guided me through the occasional turbulent time. In particular, I thank Dr Inoni Betuela, Dr Ken Boone and his wife Lisa, Samson Akunaii, Anderson Puwa and Henry Pako. The members of the Kuru Project, past and present, have made a significant contribution to the success of the Project, and though I will not name them individually, it is important that their contributions be acknowledged in these proceedings.

The collaboration of the local communities in 22 linguistic groups has allowed us to collect over 4000 blood samples, other specimens and interviews. Their participation has been generous and, without it, our knowledge of prion diseases would be more limited. The Papua New Guineans who attended the meeting represented all those people from Papua New Guinea who have assisted in the research on kuru over many years.

Kuru is no longer a priority for the people affected: their health concerns are now focused on diseases that are no longer common in wealthy countries and on the emerging AIDS epidemic. For the duration of the project, we have tried to provide medical care where possible and to source external funding for development projects.

The team's work is part of the ongoing research started by Vincent Zigas and Carleton Gajdusek in 1957 and there are many others, including some of those attending the meeting, who were the pioneers of kuru research. Much of the current research success has been dependent on the goodwill and integrity shown by the pioneers to the people of the kuru-affected region and to the long-term relationships established early on with those populations. We have tried as much as possible to follow the example set by the early research workers.

I also thank John Collinge, Frank Cooper and the staff at the MRC Prion Unit. There have also been other members of the MRC Prion Unit who have assisted in the fieldwork: Edward McKintosh, Skip Jackson, Toby Bentley, Edward Lagan and, most recently, Dafydd Thomas, who assisted with the clinical examination of patients.

I thank the Wellcome Trust for initially funding the project and, more recently, the Medical Research Council for funding support, the Life Neurological Trust for making a generous donation to the local preschools and the British High Commission in Port Moresby for funding the Okapa District Water Supply Project.

Finally, I would like to emphasize that the PNGIMR has contributed, and continues to contribute, to medical research at an international level, supports the annual Medical Symposium in PNG and publishes the *PNG Medical Journal*. It is important that the world sees Papua New Guinea in the positive light that it deserves.

